# Supplementary material for: Are carbon emissions trading and green financial instruments synergistic? -Comprehensive quantitative research based on content analysis
Source: PLoS One. 2024 Mar 7;19(3):e0298601. doi: 10.1371/journal.pone.0298601 (PMC10919601; doi:10.1371/journal.pone.0298601)
Supplement: S1 Data — (ZIP) [file pone.0298601.s001.zip › Data/地级市Z_F工作报告.zip!/╣≤╓▌/┴∙┼╠╦«╩╨/└┤╫╘╣1⁄2╓┌║┼í╛┬φ┐╦╩2╛▌═°í┐_2003.docx]

各位代表：

　　现在，我代表市人民政府，向大会报告过去一年的工作以及2003年的工作安排意见，请予审议，并请各位政协委员和列席人员提出宝贵意见。

2002年政府工作回顾

　　2002年是新一届市政府在上届市政府奠定的良好基础上继续前进的一年。一年来，在省委、省政府和市委的领导下，在市人大、市政协的监督、支持和帮助下，我们坚持以邓小平理论和“三个代表”重要思想为指导，按照“抢抓一个机遇，发挥三大优势，推进六大工程，实施八项任务”的总体思路和工作部署，解放思想，实事求是，与时俱进，开拓创新，通过全市上下的共同努力，克服了固定资产投资下滑和农业受灾两大不利因素的影响，圆满完成了市五届人大一次会议确定的目标任务，经济社会发展取得了较好成绩。

　　――经济增长速度加快。全年国内生产总值达到101.11亿元，增长10.0%，是“九五”以来增幅最大的一年。其中第一产业增长1.4%；第二产业增长12.2%；第三产业增长10.5%。全市财政总收入完成11.6亿元，增长21.8%，其中，地方财政收入完成6.52亿元，增长20.6%。

　　――结构调整力度加大。以实施“农业六大工程”为突破口，加快农业结构调整步伐。优质农产品比重有所提高，蔬菜、茶叶、烤烟、水果、牧草等种植面积扩大，产量增加，效益提高。畜牧养殖业呈现良好发展势头，产值增长7.75%，肉类产量增长9.5%。乡镇企业在困难中前进。农业产业化经营有所进步，涌现了一批种养业专业村和增收致富典型，部分农民行业协会组织开始发挥作用。继续坚持“三改一加强”方针，着力推进工业结构调整。企业技改力度加大。多渠道筹措1.19亿元资金对部分骨干煤矿进行改造。加大了壁式采煤等正规采煤方法推广和瓦斯自动监测系统建设的力度，140对矿井实现了正规采煤法，120对矿井安装了瓦斯自动监测系统。开展了“工业学水钢”活动，企业内部管理得到加强。强化了对重点行业和企业的协调服务，工业经济运行环境进一步改善。工业增加值同比增长20.7%，是1998年以来增幅最高的一年，拉动国内生产总值增长9.1个百分点。非公有制经济发展加快，注册资金、从业人员、实现税收增势较好，实现税收2.37亿元，同比增长20%，占全市税收总额的19%。服务业发展水平不断提高。商贸流通经营进一步优化，房地产、邮电通信、中介服务等行业发展迅速，旅游业发展有新的突破。

　　――基础设施建设顺利推进。全社会固定资产投资完成27.7亿元，同比下降26.8%。铁路“三线一站”全面建成并投入营运。重大建设项目顺利推进。云贵响水电站1号机组并网发电。水黄高等级公路完成工程总量的85%。市中心区防洪工程、排污工程、玉舍供水工程、城市道路等建设工程进展顺利。农村基础设施继续得到改善。林业生态工程建设取得较好成绩。城乡电网建设与改造速度加快。“十件实事”基本完成。

　　――改革开放取得新的进展。以产权制度改革为重点的企业改革继续推进。市场体系建设力度加大。农村税费扩大试点改革取得阶段性成果。开展了投资软环境治理整顿，投资创业环境有所改善，在全省整治投资环境工作评比中获得第二名。招商引资工作取得较好成效，实际到位资金11.64亿元，增长27%。

　　――城镇建设步伐加快。强化了城镇的规划建设和管理意识。双水、德坞、凤凰、荷城新区建设继续推进，市中心城区城市框架已基本形成。加大了城市户籍管理制度改革力度，城市户籍门槛进一步降低。郎岱、响水、玉舍、老鹰山等重点小城镇建设得到了加强。实施城市“亮丽工程”，加强了城市环境整治工作，城市“脏、乱、差”现象有所改善。城市基础设施建设步伐加快，城市功能逐步完善。投入城市基础设施建设资金6700万元，比上年增长18.27%。

　　――人民生活水平有了提高。加强了就业与再就业工作。共举办人力资源招聘洽谈会62次，6700人通过劳动力市场实现了就业和再就业。城镇登记失业率为4.7%。社会保障工作力度加大，市、县两级财政共投入城镇低保资金815.75万元，是上年的4.97倍；全年共发放城镇低保金3097万元，基本做到了应保尽保。“两个确保”做到了100%。城乡居民衣食住行条件有一定改善，生活水平有一定提高。消费品市场比较繁荣，全市社会消费品零售总额增长9.7%，居民消费价格总水平上涨0.1%。城镇居民人均可支配收入达到5612元，增长9.1%；农民人均纯收入达到1426元，增长4.9%。

　　――社会事业全面发展。“两基”攻坚力度进一步加大，“普九”进程加快，全市实现“普九”的乡镇（含企业）由上年底的21个增加到62个，以乡“普九”人口覆盖率由上年底的27%提高到67%。普通高中扩招突破万人大关。组建了六盘水职业技术学院并开班招生。50所“义教工程”项目主体工程全面竣工，整体工作被省评为优秀。科技工作力度加大，率先在全省设立了“知识产权专项资金”。人口、资源、环境协调发展。人口自然增长率为12.94‰。环境污染治理工作有新的进展，工业污染治理有序推进，工业废水排放达标率达72%，工业废气处理率达89%。文化基础设施不断完善，城乡医疗卫生条件进一步改善。党的民族、宗教政策进一步落实。文化、卫生、体育、广播、电视、气象、档案、地方志、对台、侨务等工作都取得了新的成绩。

　　――精神文明建设和民主法制建设不断加强。继续落实《公民道德建设实施纲要》，广泛开展城乡精神文明创建活动。依法打击各种违法犯罪活动，有效地维护了经济秩序和社会稳定。启动实施了“四五”普法规划。认真落实从源头上治理腐败的各项措施，反腐败斗争和廉政建设取得新的成绩。进一步完善廉政建设责任制，认真纠正行业不正之风。加强基层民主建设，推行了政务、村务、厂务公开。各级政府自觉接受同级人大及其常委会的监督，重视和发挥政协以及各民主党派、工商联、无党派爱国人士、人民团体的政治协商和民主监督作用，代表建议和政协提案全部办结。

　　回顾过去的一年，全市人民在富民兴市的征程中取得了令人鼓舞的成就，在加快发展的实践中焕发出催人奋进的精神力量。一年来取得的成绩来之不易，积累的经验弥足珍贵。展望未来，我们正处在凝心聚力、蓄势腾飞的大好时机。在这承前启后、继往开来的时刻，总结经验，发扬光大，意义重大。一年来工作的经验归结起来主要是：

　　――必须强化与时俱进、开拓创新的进取意识。去年，我们在创新思路、创新观念等方面进行了积极探索。着眼于区域经济的协调共进，提出了与相邻地州联手推进南贵昆经济区贵州西部经济带的构想，成功举办了“南贵昆经济区贵州西部经济带（六盘水）发展恳谈会”，并将毕节经水城、盘县至兴义高等级公路这一南北大通道作为三地州市共同促进的第一件大事来抓。强化了“大城管”观念，逐步理顺了城市管理体制，并降低了城市户籍门槛，城市管理水平有了新的提高。坚持打击与取缔并举，依法打击了非法开采煤炭资源行为，促进了安全生产形势的继续好转，在全省安全生产考核中我市名列第一。

　　――必须牢记执政为民、以人为本的服务宗旨。针对我市地质灾害隐患面大的实际，我们对全市地质灾害点进行了逐个排查，分类提出了治理措施，逐点建立了防范机制，多渠道筹集资金，对特别危险点居民进行搬迁。切实关心低收入人群的生活困难，广泛深入地开展了访贫问苦送温暖活动，进一步加深了党和政府与群众的血肉联系，赢得了群众的广泛支持。

　　――必须保持抢抓机遇、奋力拼搏的精神状态。机遇是难得的，是宝贵的，稍纵即逝。为抢抓机遇，一是进一步强化了项目前期工作,在积极向上争取资金、用好国债资金、吸引外来资金、启动民间资金的同时，市、县两级都加大了财政投入的力度。2002年，全市用于项目前期工作的经费达1630万元，其中市级财政投入达1100万元，比上年增加600万元。发耳电厂和发耳矿区前期工作加快，已列入省争取今年年底进场开展场平工程的项目。二是进一步加大了重点项目的建设协调力度。盘南电厂、野马寨电厂征地拆迁工作提前完成，基本完成场平工程。三是按照“集中财力办大事”的原则，进一步优化了基建、城建、支农、技改、科技、教育等资金的支出结构。

　　――必须弘扬脚踏实地、勇于实践的工作作风。通过深入调查研究，调整了新阶段扶贫开发思路，市委、市政府作出了进一步加快“一山一河”地区新阶段扶贫开发的决定。变群众上访为干部下访，妥善处理了原六枝矿务局破产时一次安置人员和原市建材厂职工集访问题，帮助困难群众解决了实际困难。市、区、企联合现场办公，共同探讨水钢与周边农民纠纷问题的解决思路和方案，使积压多年的问题得到较好解决。运用法律手段成功处置了红旗砖厂与周边农民纠纷引起的集访问题，收到了良好的社会效果，也为今后处理类似问题积累了宝贵的经验。采取到各县、特区、区现场办公的方式，调度工作进展情况，帮助理清发展思路，解决实际困难和问题。

　　――必须坚持建好队伍、提高素质的有效做法。五届市政府组建后，我们就对政府工作人员提出了“廉洁、高效、务实、协调”的要求。基本完成了政府机构改革。完善了政府运行机制，强化了对公务员的教育和培训。积极推进了行政审批制度改革。加强了廉政建设，对一批违纪违法人员依纪依法进行了处理。

　　我们深刻地体会到，落实富民兴市的大政方针，完成改革、发展、稳定的艰巨任务，要勇于探索、敢于攻坚、善于创新，以创新的思维、创新的胆略、创新的举措，冲破一切妨碍发展的思想观念，改变一切束缚发展的做法、规定，革除一切影响发展的体制弊端；要牢固确立率先意识、机遇意识、拼搏意识和责任意识，紧紧抓住机遇，不断推进改革开放和国民经济持续快速健康发展；要立足实际，注重实效，坚持快节奏、高效率，在抓落实上下真功夫、硬功夫；要以富民的目标凝聚人，以亲民的举措激励人，以安民的效果鼓舞人，激发全社会方方面面的积极性，把我市的社会主义现代化建设事业不断推向新的高潮。要不断加强政府自身建设，更好地适应改革开放和现代化建设的新形势，使各级政府成为人民群众真心拥护和满意的政府。

　　2002年，我市各方面工作取得了较大成就。这些成就的取得，是在中共六盘水市委的正确领导下，在市人大、市政协的有力监督、支持、帮助下，全市广大干部和各族群众认真贯彻落实中央和省的一系列方针政策，团结拼搏、艰苦奋斗的结果。在此，我谨代表市人民政府，向为加快六盘水发展作出努力和贡献的广大工人、农民、知识分子、干部、驻市人民解放军指战员、武警部队官兵、民兵预备役人员和政法干警，向各民主党派、各人民团体及其他各界人士，向关心和支持六盘水建设的境内外朋友，表示衷心的感谢并致以崇高的敬意！

　　在肯定成绩的同时，我们清醒地看到，我市经济社会发展还存在一些突出的问题和困难，主要是：粮食因灾减产在一些地方比较严重，农业产业结构调整步伐缓慢，扶贫攻坚的任务十分艰巨，农民增收难问题还未得到有效解决，严重影响了农村小康进程；固定资产投资的融资力度不够，民间投资跟进不力，投资环境特别是投资软环境还存在着许多不尽人意的地方；部分国有企业特别是地方国有企业管理不善、长期亏损的问题还比较突出；就业和再就业形势严峻，部分地区受灾群众和城市低收入家庭生活仍很困难；人口与计划生育工作两项主要指标在全省挂末；安全生产形势不容乐观，重特大事故时有发生；社会治安状况未得到根本好转，维护社会稳定工作有待进一步加强；政府职能和政府工作方式、工作作风有待进一步转变，管理水平和工作效率仍需进一步提高。这些问题，有的是长期积累下来的，有的是体制转轨和结构调整过程中难以完全避免的，有的是由于工作中的缺点和不足造成的。今后要继续采取措施，认真加以解决。

2003年政府工作的目标和重点

　　2003年是全面贯彻落实党的十六大精神的开局之年，也是全面建设小康社会的起步之年，做好2003年政府的各项工作，意义十分重大。今年政府工作的总体要求是：以邓小平理论和“三个代表”重要思想为指导，认真贯彻党的十六大、十届全国人大一次会议和省第九次党代会精神，以全面建设小康社会为目标，按照“四个三”要求，努力走跨越式发展路子，抢抓西部大开发、新阶段扶贫开发的机遇，全面提高对内对外开放水平，切实加强农业的基础地位，大力发展农业和农村经济，努力扩大投资规模，大力推进经济结构战略性调整，努力加快农业产业化、新型工业化、城镇化进程，促进国民经济持续快速健康发展，实现速度和结构、质量、效益相统一；正确处理改革发展稳定的关系，深化国有企业改革，完善社会保障体系，切实做好就业和再就业工作，努力提高城乡人民生活水平，保持社会政治稳定；大力实施科教兴市和可持续发展战略，推进各项社会事业全面进步，促进人口、资源和环境协调发展；切实加强民主法制建设、精神文明建设和党的建设，促进社会主义物质文明、政治文明、精神文明协调发展；加强政府自身建设，切实转变政府职能，进一步开创富民兴市的新局面。

　　主要预期目标是：国内生产总值增长9%以上，力争达到10%。全社会固定资产投资完成35亿元，增长26%。全市财政一般预算收入（同口径）增长10%以上。城镇居民人均可支配收入增长6%左右。农民人均纯收入增长5%左右。居民消费价格平均指数101%左右。人口自然增长率控制在13‰以内。城镇登记失业率控制在5%以内，促进就业人数达到2.1万人左右。

　　围绕完成上述预期目标，重点要抓好十个方面的工作：

　　一、扩大固定资产投资规模，加快推进经济发展

　　把投资作为拉动经济增长的主要因素，全面启动有效需求，是我市一项长期的任务，是政府工作的重中之重。要广开渠道，尽力扩大固定资产投资规模。

　　切实做好项目前期工作。坚持把项目作为争取国家投资、招商引资的关键，进一步抓紧抓好项目前期工作。今年市级财政预算内拟安排的项目前期工作经费达到1400万元，重点用于煤电、交通、城市基础设施项目的前期工作。各县也要切实调整财政支出结构，加大对项目前期工作经费的投入，以确保4个县、特区、区的前期工作经费投入达到1000万元以上。积极探索建立项目前期工作基金，并制定项目前期工作经费的筹集、管理、使用、回收办法。积极探索项目前期工作市场化的路子。切实加大煤电、交通、水利、水电、生态建设、扶贫开发、环境治理等项目的前期工作力度，加快发耳电厂、六枝电厂技改、盘东电厂等项目的前期工作，加快比德向斜东南翼、土城向斜东翼的勘探，继续做好煤化工产业发展的有关前期工作。加快推进六盘水支线机场的前期工作，完成预可研。联手推进毕节经水城、盘县至兴义高等级公路建设的前期工作。加快白鹤沟水库供水工程、六枝城区供水等项目的前期工作。在搞好水资源调查的基础上选定市中心城区后续供水水源并积极开展前期工作，为解决2008年后市中心城区的缺水问题做好准备。

　　加快推进投融资体制改革。在抓住国家继续发行国债的机遇、努力争取更多的国债资金支持的同时，必须把扩大融资、吸引外资和促进民间投资作为一项重要的战略措施，尽快形成以市场推动为主的固定资产投资增长机制。要根据项目的性质和特点，采取多种投融资方式扩大资金来源，促进新项目的建设。要进一步改善投资环境，为投资者提供简明、规范、高效、优质的服务，在市场准入、投融资、取费、用地、用电、供水等方面营造公平竞争环境，使非公有制经济发展、招商引资工作取得更大的成效。进一步增强政府信用，健全担保体系，争取金融部门对地方经济发展更大的支持，形成“贷―还―贷”的良性循环。今年，市财政预算拟压缩基本建设和城市建设等方面财政资金直接投入，变直接投入为间接投入，基本建设和城市建设等所需资金主要通过财政预算安排还本付息资金争取银行贷款解决。今年拟争取银行贷款2亿元用于基本建设和城市建设，财政预算拟安排还本付息资金4500万元。

　　加强项目建设和管理。以路、水、电为重点，加快基础设施建设。开工建设红果至威舍公路和六枝至纳雍公路等县际油路，加快俄脚至发耳公路的前期工作并争取开工建设。完成玉舍供水工程主体工程建设，开工建设明硐水库，加快病险水库治理。完成第二期农村电网改造工程，提高“一户一表”率。实施城市电网改造工程。认真落实项目法人责任制、招标投标制、工程监理制、合同管理制，严格质量、工期、投资“三控制”。

　　二、千方百计做好农业和农村工作，加快农民脱贫致富步伐

　　我市全面建设小康社会的重点在农村，难点也在农村。要按照中央关于“更多关注农村、关心农民、支持农业”的要求，以农业和农村经济结构调整为主线，以增加农民收入为基本目标，以发展优质、高产、高效、生态、安全农业为核心，抓好“五个一百”，实现“五个突破”。针对我市农业生产中解决温饱、调整种养结构和实施农业产业化三个阶段并存的实际，进行分类指导，“三管齐下”，整体推进农业和农村经济的发展。

　　大力推进新阶段扶贫开发。抓好以“一山一河”地区和贫困乡、极贫村为重点的新阶段扶贫开发工作。今年拟从各种财政专项资金中切块投入“一山一河”地区的扶贫开发，市级切块资金的总规模要达到850万元，加上县、乡和其他渠道的资金，要确保总投入达3000万元以上。按照“以规划定方向，以方向做项目，以项目配资金”的方式，重点加大农村水、电、路及沼气、通信等基础设施的建设力度，加快教育、文化、卫生等事业的发展。高度重视农田基本建设，抓好4.6万亩坡改梯建设。进一步加强土地管理，切实保护耕地。正确处理好吃饭和建设的关系，在确保生态平衡的前提下，加大土地整治力度，努力实现耕地占补平衡。坚持“蓄、引、提”并举，突出解决好农业缺水问题。启动水城县发耳片区万亩旱地灌溉工程的前期工作，开展以解决旱地灌溉为主的中小水库群的蓄水工程规划和项目前期工作，争取部分工程项目立项建设，为解决全市农田的工程性缺水探索路子。继续实施“渴望工程”，解决农村10万人的饮水困难。加大救灾救济投入，切实解决好困难群众的生产生活问题。抓好地质灾害防治和帮助解决生存条件恶劣地区群众的生产生活问题，完成2000户生存条件恶劣、地质灾害受胁区群众的搬迁。大力开展“农业学色绿”活动，继续抓好100个“学色绿”示范村和100个极贫村重点帮扶活动。以提高小额扶贫贷款回收率为主线，落实基层单位和党政干部的责任，扩大小额扶贫贷款发放范围，大力推进以贷脱贫致富。高度重视农村剩余劳动力输出，有计划、有针对性地开展技能培训，增强农村劳动力的就业能力，扩大输出规模，提高输出质量，拓宽农民增收渠道。

　　着力抓好农业结构调整。毫不放松粮食生产，力争粮食产量恢复到72万吨以上。引导农民继续加大种植业结构调整力度，扩大优质作物品种的种植面积。坚持“制、繁、引、串”并举，切实解决种子退化问题，因地制宜推广良种良法。把加快畜牧业发展作为农业结构调整的突破口和增加农民收入的重要渠道，下大力抓好。全面加强品种改良、饲草饲料、疫病防治三大体系建设。采取加大财政投入、强化技术服务、搞好市场引导等有力措施，选择100个畜牧业发展条件较好的村作为“畜牧百强村”进行重点扶持。以市场为导向，合理调整烤烟种植品种和生产布局，大力推广先进的烤烟种植和烘烤技术，确保上等烟叶比重达到30%。积极争取省有关部门的支持，逐步理顺烤烟、卷烟的生产和管理体制。切实抓好100个增收示范典型的推广，并再新增一批增收示范典型，推动农业结构调整。

　　加快推进农业产业化进程。实施龙头带动战略，大力推进农业产业化经营。加快一批农业产业化龙头企业的建设，力争立项实施云贵高原贵州盘县优质肉牛产业化建设项目；积极发挥供销合作社服务“三农”的作用，积极引导乡镇企业参与发展农产品加工、保鲜、贮藏、运输等行业；积极引导民间资本、工商资本发展农副产品加工业，加快各类农副产品批发交易市场建设，努力构建贸工农、产加销一体化经营体系；坚持“农民自愿办、农民自己管、农民自身受益”的原则，积极培育、扶持、规范各种农村专业协会和合作经济组织，完善其服务功能，提高农民进入市场的组织化程度。发挥我市农业资源的比较优势，加快建设优势突出的农产品生产基地。积极发展生态农业、城郊农业和观光农业，使之成为新的农业增长点。大力加强农业服务体系和质量标准体系建设，力争在我市建立农产品检测中心和标准化生产综合示范区。

　　三、加快经济结构调整步伐，提高经济增长的质量和效益

　　按照走新型工业化道路的要求，加快工业结构调整，促进产业优化升级。切实抓好盘南电厂、野马寨电厂、响水煤矿等重大电力项目和大型煤矿项目的实施。推进响水、发耳、格目底、松河等井田的渐进开发。坚持内外结合、煤电联营，组建新公司开发响水煤矿、发耳矿区。认真执行《市人民政府关于新建扩建项目税收实行分级管理的意见》，充分调动市和县新建项目的积极性。把加强工业技术改造放到突出位置。积极争取国家国债专项计划，落实技改项目贴息，扩大信贷规模，保证重点项目顺利实施。各级财政要安排资金，用贴息方式引导企业资金和信贷资金投入技改。今年市级财政投入的技改资金1200万元，重点支持水城水泥公司日产2000吨熟料新型干法生产线、六盘水啤酒有限公司“三改十”一期工程等技改项目建设。支持有证煤矿加大安全生产方面的技改投入，重点支持200对力争257对有证煤矿安装瓦斯自动监测系统。新建和改扩建骨干煤矿60对以上，争取60%以上的煤矿实现正规采煤法。继续做好协调服务工作，确保云贵响水电站发电正常运转。与此同时，积极扶持发展面广量大的中小企业。大力开展“工业学水钢”活动，采取从市县行政事业单位和企业选派一批干部到大企业挂职、帮助中小企业理清发展思路、部门既重管理更重服务等有效措施，帮助企业改进机制、转变观念、完善工艺、开拓市场，通过改制、改组、联合、兼并等方式，千方百计把一批中小企业盘活。

　　大力发展非公有制经济，是我市经济结构调整的重大任务。今年要以营造环境为主题，认真开展“发展非公有制经济环境整治年”活动。抓住落实政策是否到位、部门服务是否改善、行政执法是否合法三个重点，对发展非公有制经济的环境情况进行一次全面调查，对照“五给”、“五不限”的要求，找准差距和症结，在此基础上出台进一步加快非公有制经济发展的政策措施并认真加以落实。针对我市非公有制企业发展中存在的经营环境问题和经营理念问题，通过各级政府的“两帮两促”，即帮助非公有制企业创造良好的生产经营环境，促其正常经营；帮助非公有制企业转变发展观念、适应市场变化，促其发展壮大。加大治理“三乱”工作力度，认真查处“吃、拿、卡、要”等行为，切实维护非公有制经济的合法权益。用好用足中小企业担保资金，加大对中小企业和私营个体经济的金融信贷支持力度。

　　加快发展服务业和旅游业。努力培育具有现代经营理念、现代经营业态和现代经营环境的商业物流产业。大力推进国有商贸企业改革，提高商贸企业的竞争能力。认真实施《市委市政府关于进一步加快旅游业发展的意见》，抓紧做好旅游发展和景区建设规划，加强旅游基础设施建设，加快旅游业的发展。切实增强既重资源更重市场的观念，面向本埠游客重点开发麒麟公园、玉舍森林公园、南开风景区阿勒河景区、?咪期度假区等近郊旅游，在此基础上逐步推进六枝??江景区、盘县大洞竹海、古银杏景区等旅游资源开发。切实做好第二次三产普查工作。

　　大力推进区域经济发展，切实增强区域经济的辐射和带动作用。紧紧抓住国家实施西部大开发中建设“南贵昆经济区”的机遇，与毕节地区、黔西南州继续推进贵州西部经济带建设。认真研究“边缘政策”，选择有条件的地方建立专业市场或工业、贸易园区。与此同时，市境内自然条件相近、地缘相邻、发展水平相当的地区，要打破行政区域界线，大力发展沿交通主干线的通道经济、沿工矿带的城镇经济、沿低热河谷区的特色农业经济等区域经济。支持盘县、钟山区争取进入全省第二轮建设经济强县，启动全市第二轮建设经济强乡（镇）工作。启动6个小康乡（镇）、12个小康村建设。

　　四、强化城镇规划、建设和管理，着力提高城镇化水平

　　根据经国务院同意、由建设部批准的《贵州省城镇体系规划》，瞄准把市中心城区建成大城市和把六枝平寨、盘县红果建成中等城市的目标，按照“做大、做强、做好、做美”的要求，以加快市中心城区建设为重点，同时大力推进重点小城镇建设的城镇化思路，大力实施城镇化战略。狠抓规划、建设、管理三大环节，进一步完善科学高效的城镇规划管理体系、多元化的城建资金筹资体系、市场取向的公用设施运行体系、严格长效的城市综合管理体系等机制，努力塑造城市新形象，加速推进城镇化。

　　继续加快市中心城区双水、凤凰、德坞、荷城等新区的开发建设。同时，在2002年降低城镇户籍门槛的基础上，在教育、就业、第三产业等方面进一步降低城市门槛，逐步增大城市人口总量。强化规划的龙头地位作用，进一步加强规划的集中统一管理。坚持规划管理的法制化、科学化、民主化，积极实行规划的公示、听证制度，提高规划的透明度和决策水平。抓好市中心城区近期建设规划的调整，完成10个小城镇建设规划。统筹规划和加快市中心城区道路、防洪、排污、供水、供电、供气、垃圾处理等城市基础设施。全面建成市中心区防洪一期工程、排污一期工程，开工建设龙井路立交桥和水西南路立交桥。加快市中心区垃圾填埋场的建设进度，开工建设排污管网配套工程、供水管网二期工程。加快市政基础设施投资管理体制改革，吸引各方面资金投资公益性基础设施建设。在调研、论证、听证的基础上，进一步理顺城镇供水价格体系，适时开征垃圾处理费。加大平寨、红果的开发建设力度。继续推进重点小城镇建设，使之与市中心城建设相配套、相协调，努力形成合理布局、功能互补的现代化城镇体系格局。

　　进一步强化经营城市的理念，通过激活土地、资金、人才、信息等生产要素，壮大城市经济。建立健全城市建设投入、回报、偿还、再投入的良性循环机制。推行土地收购储备制度，政府垄断土地一级市场，严格实行经营性项目土地使用权公开挂牌、招标、拍卖出让，努力实现城市土地收益的突破。积极探索广告经营权、冠名权出让转让的有效方式。切实关注房地产业发展动向，防止出现过热现象和无序发展。认真研究激活房地产二级市场的措施并切实加以落实。

　　按照“注重基础、强化整治、立足长效、全面提高”的要求，加强城市管理。加快理顺城市管理体制，积极实施以块为主、分级管理的“两级政府、三级管理、四级网络”城市管理体制。认真落实已出台的城市管理办法。加大城市管理改革力度，积极创造条件，稳步推进城市管理行政执法改革，逐步解决多层次执法和多头执法的问题。适时推进环卫、绿化、公交等市政公用行业的改革，努力实现投资多元化、运营市场化、发展产业化。与此同时，积极推进社区建设，上半年完成钟山区一个街道办、六枝特区一个社区的社区建设试点工作，下半年逐步推开。

　　继续实施“亮丽工程”，美化城市环境。在巩固去年钟山路实施“亮丽工程”工作成果的基础上，加大实施力度，扩大实施面，推进灯光工程、绿化工程、建筑外观工程、市政设施工程、环境整治工程，力争年内城市环境、生活质量、城市形象再向前迈进一步。全面清理市中心城区环城绿化带工程实施情况，进一步加大投入，强化管护责任，力争绿化工程在5年内初见成效，10年内基本建成。

　　五、加快改革开放步伐，提高对外开放水平

　　以国有企业改革为重点，切实抓好产权制度改革和完善法人治理结构两大关键环节，着力推进市场取向改革向多领域、多行业拓展，努力形成各行各业齐抓改革、锐意创新的新局面。继续抓好国有企业改革，建立和完善企业内部三项制度。按照中央和省的部署，稳步推进国有资产管理体制改革，建立健全管资产和管人、管事相结合的国有资产管理体制。加快国有经济战略性调整，推进国有企业改革转制。对于已经改制的大中型企业，要通过建立规范的法人治理结构，形成责权统一、协调运转、有效制衡的运作机制。认真贯彻落实《中小企业促进法》，建立中小企业发展基金，进一步放开搞活国有中小企业。长期稳定党在农村的基本政策。继续完善农村税费改革政策，巩固改革成果，减轻农民负担。继续深化医疗、保险制度改革。加大药品采购的招投标力度。积极开展新型农村合作医疗保障体系改革试点，努力探索筹资、运行、管理机制。继续推进粮食购销市场化改革。加快深化供销合作社改革进程，逐步完善农村市场体系。大力培育矿业权市场，规范采矿权行政审批，启动实施采矿权招标拍卖、挂牌出让。积极推进财政改革，进一步扩大部门预算执行范围，切实落实“收支两条线”有关规定。严格执行《政府采购法》，依法规范政府采购行为，逐步扩大政府采购范围，提高采购资金节约率。推进国库集中收付试点工作。在规范运作市公益信用担保公司的基础上，积极探索建立我市的国有资产运营公司或国有资本参股的资产运营公司，努力实现国有资产的保值增值。积极探索资本运营方式，逐步推进国有资本的整合和盘活。按照政事分开的原则，坚持市场取向，坚持社会事业公益化与市场化、产业化相结合的发展方向，积极推进事业单位的用人、分配制度改革。

　　继续严厉打击制售假冒伪劣商品的不法行为，大力开展集贸市场、加油站、旅游市场、建筑市场等专项整治。坚持标本兼治，建立健全市场准入制度，加强法制建设，严格执法。深入开展规范西电东送电煤基地矿业秩序工作，按照省政府的统一部署对规划的和拟规划的西电东送电煤基地内的煤矿进行清理，确保矿业秩序的进一步好转。坚持依法治税，维护税法尊严。切实整顿税收征管秩序，继续做好税收专项整治工作，严厉打击偷、逃、骗、抗税行为，坚决杜绝越权减免税。加强对煤炭产品税收的征收管理。

　　树立环境是生产力和竞争力的新观念，把改善投资软环境作为招商引资的基础性工作来抓。进一步加大招商引资力度，不断扩大全市利用外资规模，提高利用外资水平。推行定向招商和定点招商，实行重点招商项目领导负责制。认真总结去年开展投资软环境综合整治的经验，针对存在的突出问题，进一步加大整治力度，减少投资审批程序，提高办事服务效率。加强对投资项目的跟踪服务工作，及时解决投资业主的困难，树立良好的招商引资形象。编制行政机关服务手册。实行部门行政执法时外商投资服务中心参与制，实行行政收费一卡制。全面清理各级各部门出台的难以兑现的违规承诺。进一步加大对外宣传工作力度，提高六盘水知名度。

　　六、切实做好就业和再就业工作，进一步完善社会保障体系

　　要把促进就业、加强社会保障作为事关全局的重要工作来抓。坚持“劳动者自主择业、市场调节就业、政府促进就业”的方针，通过大力发展劳动密集型产业、促进劳务输出、采取灵活多样的就业方式等措施，努力增加就业岗位，促进城镇2.1万人实现就业。全面落实小额信贷担保、税费减免、社会保险补贴等方面促进再就业的优惠政策。继续开展再就业援助活动，重点做好“4050”人员的再就业帮扶工作。加大对下岗失业人员的培训力度，认真抓好对再就业者的“订单培训”和对自主创业者的培训，完成万人以上培训任务。高度重视高等院校扩招后毕业生的就业问题，超前研究，妥善解决。

　　继续巩固“两个确保”100％的成果，搞好“三条保障线”的衔接，对协议期满暂时无法解除劳动关系的下岗职工，继续运用现有各类筹资渠道保障其基本生活。各级财政要优先安排“两个确保”、“城市低保”和再就业资金的投入。要按照市、县两级财政各承担配套低保资金15%的要求，足额纳入本级财政预算安排。规范低保的申请、审批、发放和监督工作，加强对纳入低保范围人员的动态管理。

　　切实关心困难群众和灾区灾民的生产生活，认真做好受灾地区和灾民的生产自救、农业税减免及救济等工作。高度重视城市贫困居民的住房困难问题，年内启动廉租房建设。加强社会救助和帮困工作，不断完善社会救助制度和运作机制。关注弱势群体，加强社会福利和残疾人事业。开工建设市社会福利中心。大力发展社会慈善事业，开展社会互助救济、帮困扶贫，逐步完善社会救助保障体系。进一步加快老龄事业发展，努力做到“六个老有”。

　　七、实施科教兴市和可持续发展战略，加快社会事业发展

　　坚持把教育放在优先发展的战略地位，大力开展“重教兴教年”活动，全面发展以“两基”攻坚为重点的教育事业。采取坚决措施，实现30个乡（镇）的“普九”。抓住国家继续实施“中小学危房改造工程”和“第二期义教工程”的机遇，认真研究解决中小学布局不合理的问题。采取有效措施，整合市中心城区教育资源，尽快解决初中上学难的问题。高度重视并切实解决中小学教师缺额问题。加大教育经费的投入，加强管理和使用，严禁将教育费附加、学杂费等教育资金用于平衡财政或挪作他用，维护正常的教学秩序。开展回访复查活动，防止“普六”、“普九”成果滑坡，严格控制中小学辍学率。加大高校贫困学生助学贷款力度，努力解决其入学困难。鼓励、支持、引导、规范社会力量办学。深化教育改革，加强职业教育、普通高中教育和高等教育。努力办好六盘水职业技术学院和贵州师范大学六盘水学院，做好六盘水师范高等专科学校与六盘水师范学校合并的前期工作，完成六盘水职业技术学院建设规划并开工建设综合教学楼。

　　坚持把科学技术作为经济社会发展的重要推动力，加大科技投入，加快科技进步和技术创新。广泛引进、消化和吸收先进技术，加快科技成果向现实生产力的转化。加大产业结构调整的科技试验示范力度，积极采用高新技术改造和提升传统产业。加大专利申请及转化的支持力度，鼓励自主创新。重视医药工业的发展，加快六枝特色医药工业园区建设的前期工作，多渠道筹措资金进行建设，营造好投资软硬环境，吸引医药企业入园发展。启动建设市级办公业务资源网，加快推进电子政务建设，逐步在各行业推广先进适用的信息技术。

　　高度重视并下大力抓好人口与计划生育工作，控制人口数量，提高人口素质，切实扭转去年我市人口与计划生育工作的被动局面。强化计划生育工作党政一把手负责制，坚决兑现“一票否决制”。切实加大财政投入。市级财政投入拟在去年的基础上增长57%，人均要增长55%，县、乡必须相应加大投入。采取整顿队伍、强化培训等措施，着力提高计划生育工作队伍的整体素质。高度重视基层基础工作，重新审视“村为主”工作水平，按照“切块分割，分类建设，强攻薄弱，全面推进”的思路，加强“村为主”建设，防止“村落空”。加强计划生育工作法制建设，提高依法行政水平，避免简单粗暴。抓好妇检工作，提高妇检质量，实现“关口”前移，控制计划外二孩出生，杜绝计划外多孩出生。认真落实部门责任制，切实加强流动人口计划生育管理，努力形成综合治理、齐抓共管的工作格局。

　　加强生态环境建设和环境保护。要加大工作力度，认真抓好以退耕还林为重点的林业生态建设，切实加强森林资源的保护和管理，做好退耕还林的定权发证工作。重点抓好100个生态小康文明村建设。积极准备“一江两河”污染治理项目，争取纳入国家和省的盘子，通过实施项目推进污染治理。巩固“五小”企业关闭和“两土”取缔成果，防止回潮反弹。继续做好文化、体育、卫生等工作。

　　八、认真做好维护稳定的各项工作，确保社会政治稳定

　　要深入开展各种不稳定因素的排查调处工作，预防和妥善处置由人民内部矛盾引发的群体性事件。深入困难企业和农村，全面掌握影响稳定的苗头和各种不安定因素，变群众上访为干部下访，努力把各种矛盾和问题解决在内部、解决在基层、解决在萌芽状态。切实转变作风，加强信访工作，采取行政、经济、法律等各种手段和思想政治工作方法，抓好各种矛盾、纠纷的处置和疏导。按照“进度服从稳定”的原则，积极配合省有关部门搞好杉树林铅锌矿、老鹰山煤矿的关闭破产工作。

　　各行各业都要高度重视安全生产工作。把认识保安全、责任保安全、检查保安全、整改保安全、技术保安全有机统一起来，防治结合、标本兼治、综合治理。切实加大对煤炭生产、道路交通、防火防爆、危险物品等安全事故多发行业和领域的安全管理。继续坚决打击与取缔非法开采煤炭资源的违法行为。坚决克服安装瓦斯自动监测系统后万事大吉的麻痹思想，认真排查有证矿井的安全隐患，坚持“发现隐患、暂扣证照，停产停运，派人驻点，督促整改”的做法，切实消除安全隐患。对爆炸物品安全管理，要落实到运、储、领、用等各个环节。

　　加强社会治安综合治理，进一步提高对社会治安的防控能力，切实增强人民群众的安全感。继续深入开展“严打”整治斗争，依法严厉打击毒品、经济、刑事犯罪和“法轮功”邪教活动，加大对偷牛盗马、入室抢劫等犯罪团伙的打击力度。切实加强隐蔽战线斗争，严密防范和依法打击敌对势力的破坏活动。

　　九、加强精神文明和政治文明建设，促进社会全面进步

　　坚持“两手抓、两手都要硬”的方针，进一步加强精神文明建设和政治文明建设，为改革开放和经济建设提供强有力的思想保证、智力支持和精神动力。深入开展群众性精神文明创建活动和文化、科技、卫生“三下乡”活动。加快市文化科技中心和乡镇精神文明活动中心的建设步伐。重视村镇规划建设，切实推进农村“五改”。认真贯彻《公民道德建设实施纲要》，加强社会公德、职业道德和家庭美德教育，深入开展爱国主义、集体主义、社会主义思想教育，逐步提升市民现代文明素质，积极塑造健康向上的人文环境。大力实施“四五”普法教育，增强全市人民特别是各级干部的法制观念和依法办事能力。自觉主动地接受人民代表大会及其常务委员会的法律监督、工作监督以及政协的民主监督。进一步推行和规范厂务公开、村务公开，扩大基层民主。加强基层组织建设，努力提高村（居）委会干部报酬。开展诚实守信、依法经营的教育活动。加强国防教育和国防动员工作，深化民兵工作改革，加强预备役和应急分队建设，加大人防战备工作力度。广泛深入地开展争创双拥模范城（县）活动，巩固和发展军政军民团结。继续做好民族、宗教、对台、侨务、档案、地方志和年鉴等工作。

　　十、加强政府自身建设，切实转变政府职能

　　要紧紧围绕提高行政水平和服务质量，以推进行政效能建设为着力点，切实转变政府职能，改进工作作风，建立起行为规范、运转协调、公正透明、廉洁高效的行政管理体制。按照国家和省的部署，深化政府机构改革，构筑政府、企业、市场的新型关系，切实把政府职能转变到经济调节、市场监管、社会管理和公共服务上来。积极创建学习型机关，人人争做勤奋学习、善于思考的模范，解放思想、与时俱进的模范，勇于实践、锐意创新的模范。加大培训力度，不断提高公务员素质。进一步规范行政执法行为，加强行政执法监督工作，实施行政过错责任追究，严肃查处执法中的违法违纪问题。进一步加强廉政建设，切实抓好反腐倡廉，坚决纠正部门和行业不正之风，努力从源头上、机制上、制度上预防和治理腐败。严肃财经纪律，认真落实党政主要领导干部任期经济责任审计制度和《六盘水市党政主要领导干部任期经济责任审计年度自查备案办法（试行）》。加大政务公开工作力度，增强政府工作透明度，自觉接受人民群众和新闻媒体对政府工作的监督。牢记“两个务必”，纯洁党风政风。切实改进领导方式和工作作风，大力精简会议和文件，提高工作的质量和效率。深入基层、深入群众，倾听群众呼声，关心群众疾苦，及时解决群众关心的热点和难点问题，增强执政为民的公仆意识。切实办好2003年“十件实事”，真正取信于民。加强经济运行监测和分析，有针对性地采取措施，提高经济运行的质量和效益。认真做好财税工作，继续大力增收节支。切实调整支出结构，按照“集中财力办大事”的原则，保工资、保机构运转、保重点支出，把有限的资金用好。加强对各项重要工作特别是重点项目建设进行“全流程”管理，强化督查，实行目标考核，推动工作落实。围绕推进市委、市政府决策部署的落实，切实发挥部门的职能作用，下决心解决好配合不够、推诿扯皮、办事拖拉等问题，真正形成“聚精会神搞建设，一心一意谋发展”的良好氛围。在全面建设小康社会的伟大实践中，在不进则退、慢进也是退的竞争态势中，各级政府各工作部门要意识到“放弃机遇就是失职”，牢固树立强烈的机遇意识，不断提高效率，提高质量；不断鼓劲加压，拉高起点；不断追赶一流，追求超越。要通过加强政府自身建设，转变政府职能，为积蓄更大的发展后劲、拓展更大的发展空间、形成强大的凝聚力和战斗力提供有力保障。

　　各位代表：新的时代孕育着新的机遇，新的时代充满着新的挑战。让我们紧密团结在以胡锦涛同志为总书记的党中央周围，高举邓小平理论伟大旗帜，按照“三个代表”重要思想的要求，在省委、省政府和市委的领导下，全面贯彻落实党的十六大精神，以只争朝夕、奋发有为的精神状态，艰苦创业、追求卓越的坚定信念，脚踏实地、求真务实的工作作风，团结拼搏，与时俱进，开拓进取，全面推进我市改革开放和现代化建设的宏伟大业，为共同创造我们的幸福生活和美好未来，为加快富民兴市、全面建设小康社会而努力奋斗！
